# Field Measurements of Wind-Induced Responses of the Shanghai World Financial Center during Super Typhoon Lekima

**DOI:** 10.3390/s23146519

**Published:** 2023-07-19

**Authors:** Xu Wang, Guoliang Zhang, Yongguang Li, Hu Kong, Lang Liu, Cheng Zhang

**Affiliations:** 1 State Key Laboratory of Mountain Bridge and Tunnel Engineering, Chongqing Jiaotong University, Chongqing 400074, China; xuwang@cqjtu.edu.cn (X.W.); liang_zgl@163.com (G.Z.); yongguangli@mails.cqjtu.edu.cn (Y.L.); 2 School of Civil Engineering, Chongqing Jiaotong University, Chongqing 400041, China; 3 Department of Civil and Transportation Engineering, Hohai University, Nanjing 210098, China; 18271219660@163.com; 4 Shenzhen Y. S. Mao Bridge Design Group Co., Ltd., Shenzhen 518110, China; y.s.mao@188.com

**Keywords:** super-high-rise building, dynamic characteristics, field measurement, natural frequency, damping ratio, Typhoon Lekima

## Abstract

In this paper, the wind-induced responses of the Shanghai World Financial Center (SWFC) under Super Typhoon Lekima are measured using the health monitoring system. Based on the measurements, the characteristics of vibration, including probability density distribution of accelerations, power spectra, and mode shapes are studied. The curve method and the standard deviation method are used to analyze the relationship of the first- and second-order natural frequencies and damping ratios with amplitudes and the mean wind speed. The results show the following: (1) The structural wind-induced responses in the X and Y directions have high consistencies, and the vibration signals exhibit a peak state; moreover, response amplitudes and acceleration signals disperse when the floor height increases. (2) The first- and second-order natural frequencies in the X and Y directions decrease with the increasing amplitudes and are negatively correlated with mean wind speed; the maximum decrease in natural frequency is 5.794%. The first- and second-order damping ratios in the X and Y directions increase with the increasing amplitudes and are positively correlated with the mean wind speed; the maximum increase in damping ratio is 95.7%. (3) The curve method and the standard deviation method are similar in identifying dynamic characteristic parameters, but the discreteness of the natural frequencies obtained by the curve method is lesser. (4) Under excitations of various typhoons, the mode shapes of SWFC are basically the same, and the mode shapes in the X and Y directions increase with the height and have nonlinearity.

## 1. Introduction

The rapid development of new materials, construction technologies, and design concepts has resulted in the remarkable rise of super-high-rise buildings [1]. Due to their light mass, highly reinforced material, and low damping, supertall buildings are wind-sensitive structures. Strong wind load becomes one of the dominant loads in structural design and has significant impacts on structural safety, occupant comfort, and construction costs [2]. Therefore, it is necessary to analyze wind-induced characteristics to provide beneficial references for wind-resistant design [3].

Normally, wind tunnel tests, numerical simulations, and field measurements are used to study the wind-induced responses of super-high-rise buildings. With the development of monitoring technology, field measurements have become the most accurate and reliable method for studying structural wind effects, since this method not only provides reliable information on wind effects in high-rise buildings [4] but also avoids the modelling and scaling errors implicit in numerical simulations and wind tunnel tests, such as unfavorable Reynolds number effects.

Over the past few decades, many scholars in China and abroad have carried out a series of empirical studies on the wind-induced responses of high-rise buildings. Ohkuma et al. [5], Denoon et al. [6], Kijewski-Correa et al. [7,8,9,10,11,12,13], and Yi et al. [14] conducted studies on the buildings of 68 m, 84 m, 245.7 m, 264 m, and 420 m in height, respectively. The main contents include wind-induced vibration response patterns, time-varying acceleration characteristics, and dynamic characteristic parameters, as well as the development of real-time monitoring systems and research into comfort assessment. Although the dynamic characteristics of high-rise buildings under wind action have been extensively studied by full-scale measurements, comprehensive empirical studies of wind effects on super-high-rise buildings of nearly 500 m height or even higher are still very limited, especially the dynamic characteristic parameters under strong typhoon action.

In general, the dynamic characteristics of high-rise buildings are related to their vibration amplitudes, which are usually excited by wind loads. Therefore, buildings and the wind environment can be seen as a time-varying system. Li et al. [15,16,17,18,19,20,21,22,23,24,25,26,27,28] used the random decrement technique (RDT), the Hilbert–Huang transform (HHT), and other methods to study the wind characteristics and wind-induced responses of various super-high-rise buildings in Hong Kong, Taipei, Shanghai, Guangzhou, and Shenzhen. These studies have different focuses, including the variation patterns of natural frequencies and damping ratios with amplitudes, comparisons of the measured results with wind tunnel tests, verification of wind tunnel tests, and finite element models. Zhang et al. [29] used a fast Bayesian approach to analyze the environmental vibration data of the Shanghai Center Tower at different stages of construction, identified the dynamic parameters of the structure, and compared them with the results of the finite element analysis. Fu et al. [30] compared the variations of the first-order natural frequency and damping ratio at low and high amplitudes for the Shanghai Center Tower, based on accurate wind data and the monitored data during the landfall of Typhoon Ampil. Currently, the discrete Fourier transform (DFT) method is commonly used to calculate the power spectral density (PSD) of discrete-time digital signals, but it has insufficient resolution for identifying lower frequency bands. For example, the variations in natural frequency caused by the nonlinear stiffness of structures are often at a level of 10^−3^ Hz or even less.

Although many scholars have conducted extensive research on wind vibration response measurements, the methods for extracting structural dynamic characteristics parameters for super-high-rise buildings in coastal regions have not been sufficiently discussed. Further research is required to develop suitable empirical models for wind-resistant design of structures. In this study, the wind-induced responses of the SWFC under Typhoon Lekima are explored. Firstly, the characteristics of the wind-induced responses are analyzed, including acceleration probability density, power spectrum, etc. Then, the curve method and the standard deviation method in the envelope random decrement technique (E-RDT) are applied. The amplitude dependence of natural frequencies and damping ratios and their variations with the mean wind speed are mainly studied and further compared to the findings of other scholars. Finally, the variations of overall mode shapes under four typhoons, i.e., Ampil, Rumbia, Jongdari, and Lekima, are analyzed.

## 2. Introduction to Field Measurements

### 2.1. Super Typhoon Lekima

**Figure 1:**
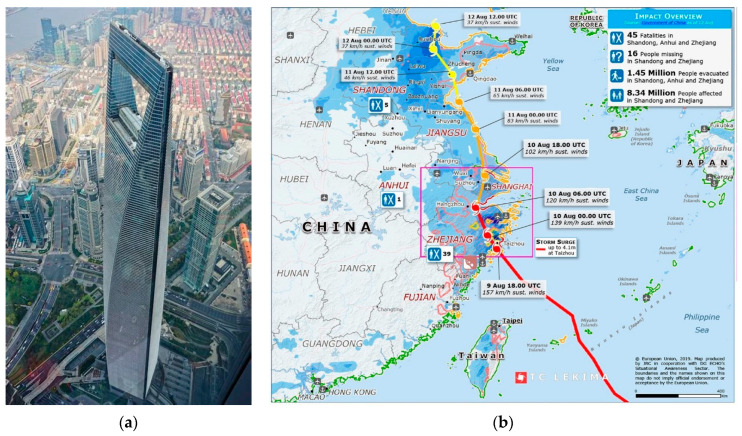
Landfall of the typhoon and building profile image. (**a**) Building profile image (image © 2023 Google; image by authors). (**b**) Path of Super Typhoon Lekima (image © 2023 Google; map produced by JRC in cooperation with DG ECHO’s Situational Awareness Sector).

The Japan Meteorological Agency named Super Typhoon Lekima at 15:00 on 4 August 2019, and it made landfall off the coast of Zhejiang at 01:45 on 10 August, with a maximum instantaneous wind speed of 52 m/s. Figure 1b depicts the moving path of Typhoon Lekima, which was characterized by a high intensity and long duration of landfall and finally caused severe disasters.

### 2.2. The Shanghai World Financial Center and Monitoring System

The Shanghai World Financial Center (SWFC) is located in the Lujiazui Financial and Trade Zone in Pudong New District, Shanghai, between longitude 121°30′8.70″ east and latitude 31°14′9.63″ north. It is a D-class landform surrounded by many super-high-rise buildings. The structure has a total height of 492 m, with 101 stories aboveground and 3 stories underground, and a total floor area of approximately 350,000 square meters. The outline of the building is shown in Figure 1a.

The SWFC uses a triple resistance system—a megaframe structure—to carry the overturning moments caused by wind and earthquakes [31]. The structure consists of giant columns, giant diagonal braces, and perimeter band trusses to improve the stiffness and integrity of the structural system. In order to suppress the vibrations of the structure under strong wind and seismic loads, two active mass tuned dampers (ATMD) were installed on the 90th floor of the SWFC. The dampers are effective in reducing building sway [32], ensuring a safe and comfortable environment for office occupants.

**Figure 2:**
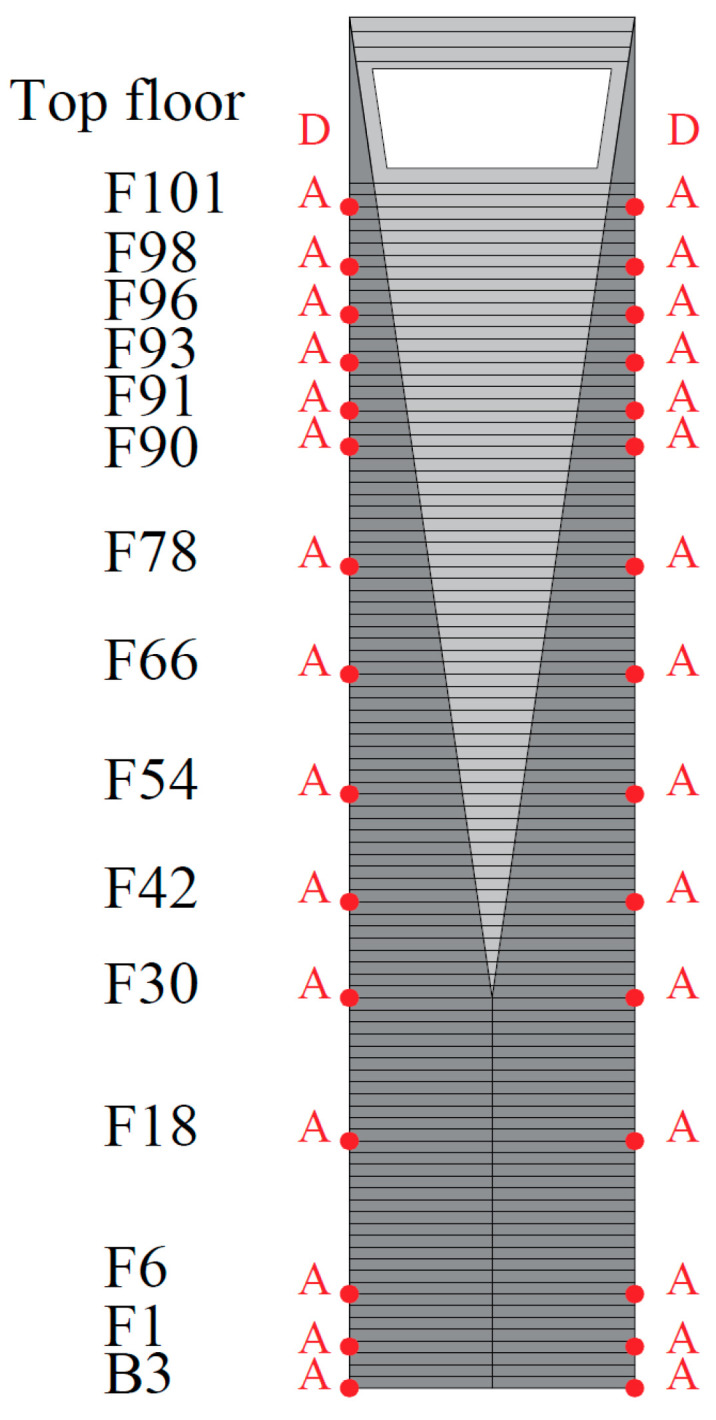
Measurement site layout of the SWFC.

**Figure 3:**
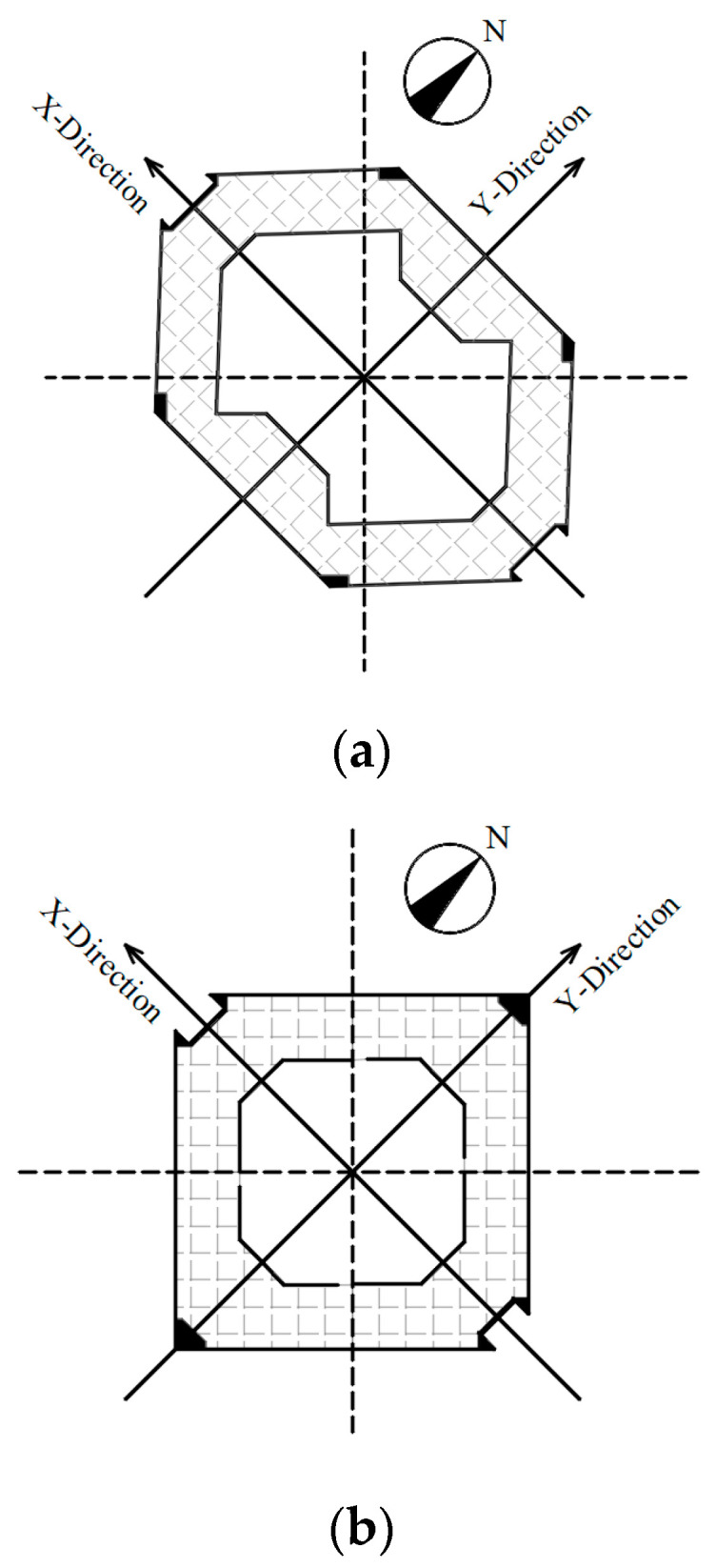
Floor plan of the SWFC. (**a**) Upper layout plan. (**b**) Lower layout plan.

To obtain the dynamic response characteristics of super-high-rise buildings under natural excitation, accelerometer sensors and anemometers were installed on the roof to form a health monitoring system. The measurement sites are installed as shown in Figure 2, where “A” is the three-dimensional accelerometer and “D” is the anemometer. The accelerometer is a TDA-33M accelerometer with a sampling frequency of 100 Hz, a sampling range of ±2 g, and a sensitivity of 1.25 V/g. As shown in Figure 3, the accelerometer takes the east–west direction of the SWFC as the *X* axis and the north–south direction as the *Y* axis. The anemometer uses 81,000 series of three-dimensional ultrasonic anemometers produced by R.M. Young, U.S.A. The working temperature is −50~+50 °C, the wind speed range is 0~40 m/s, and the measured accuracy of wind speed and wind direction are 0.01 m/s and 0.1°, respectively. The sampling frequency is 10 Hz, and the wind direction is defined as 0° in the positive north direction, and negative in the top view of the counterclockwise direction.

## 3. Data Processing

Acceleration measurements at 72 h intervals from the top of the SWFC under Typhoon Lekima were used, recorded from 0:00 on 8 August to 0:00 on 11 August 2019, in the east–west (X) and north–south (Y) directions. The curve and standard deviation methods in the envelope random decrement technique (E-RDT) were used to identify structural dynamic parameters and conduct comparative analyses as follows [33]:

(1) The curve method.

① Make modal decomposition of the original acceleration responses to obtain the timescales of the first-order and second-order modal acceleration responses *a*_1_ and *a*_2_;

② Use the Hilbert–Huang transform (HHT) to find the envelope of the first- and second-order modal timescales:

(1) $$A\left(t\right)=\sqrt{{y\left(t\right)}^{2}+\left[\frac{1}{\pi }\int_{-\infty }^{+\infty }\frac{y\left(\tau \right)}{t-\tau }d\tau \right]}$$

where *A*(*t*) is the envelope curve, *y*(*t*) is the response timescale, and *τ* is the length of one response segment;

③ Set 20 intercept amplitudes by 0.5~1.5 times the standard deviations of *a*_1_ and *a*_2_;

④ Calculate the decay curve of free vibrations based on the E-RDT method;

⑤ Fit the decay curves of free vibrations by cubic spline curve fitting to obtain the first- and second-order natural frequencies and damping ratios.

(2) The standard deviation method.

① Make modal decomposition of the original acceleration responses to obtain the timescales of the first-order and second-order modal acceleration responses *a*_1_ and *a*_2_;

② Calculate the standard deviation *S* of *a*_1_ and *a*_2_, and set *S* as the interception threshold to find the zero point of the acceleration response;

③ Calculate the decay curves of free vibrations based on the E-RDT method;

④ Fit the decay curves of free vibrations by cubic spline curve fitting to obtain the first- and second-order natural frequencies and damping ratios.

## 4. Vibration Response Characteristics of the SWFC under Typhoon

### 4.1. Mean Wind Speed and Direction

**Figure 4:**
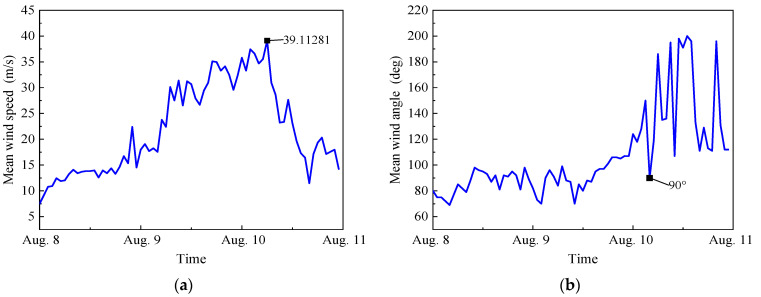
Mean wind speed and direction at the top of the SWFC: (**a**) 10 min mean wind speed, and (**b**) 10 min mean wind direction.

Figure 4 depicts the 10 min mean wind speed and wind direction angle of measurements. As shown in the figure, the mean wind speed increased slowly with time from 0:00 on 8 August to 4:00 on 9 August 2019, and then increased at a fast rate. The mean wind speed reached its peak at 05:00 on August 10, when the corresponding mean wind speed was 39.1 m/s and the wind angle was 90°, indicating that the measurement sites were closest to the typhoon at this time. After that, the average wind speed then started to drop, with the wind angle fluctuating in the range of 107° to 200°, which was caused by the changing direction of the typhoon entering the Yellow Sea.

### 4.2. Wind-Induced Vibration Analysis

**Figure 5:**
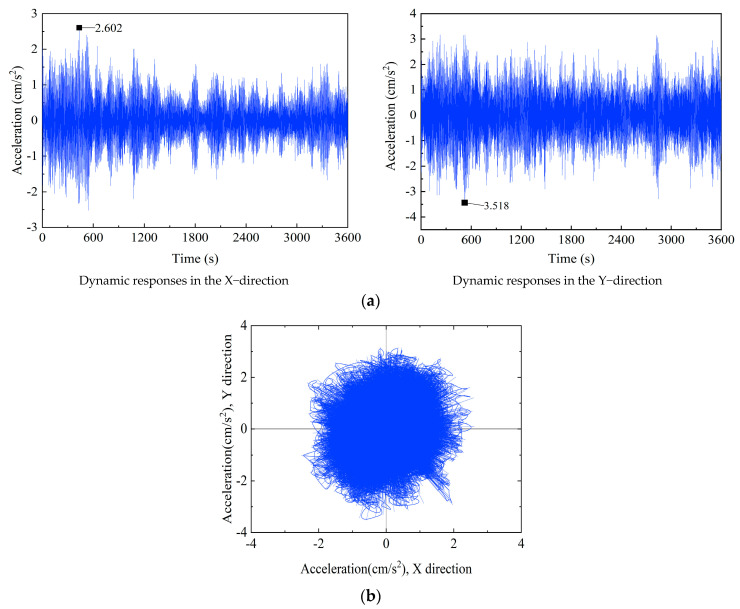
Acceleration response timescales and trajectories of the 101st floor of the SWFC: (**a**) measured acceleration response timescale at the 101st floor, and (**b**) acceleration trajectories on the 101st floor.

Wind-induced acceleration signals in the X and Y directions were also collected by the accelerometers, which are installed on 15 floors of the SWFC, at the same time. As seen in Figure 4, the peak of vibration acceleration appeared at 05:00 on August 10, so the 1 h acceleration signals near the maximum mean wind speed were chosen to plot the time history, as depicted in Figure 5a,b. As shown, the SWFC similarly vibrates in the east–west and north–south directions during the time period of interest. The maximum acceleration of 3.518 cm/s^2^ occurs in the Y direction, and the one in the X direction is 2.602 cm/s^2^. They are less than the specified value of 24.5 cm/s^2^ recommended in design codes [34,35,36], which means the SWFC meets the provisional requirements under Typhoon Lekima.

**Figure 6:**
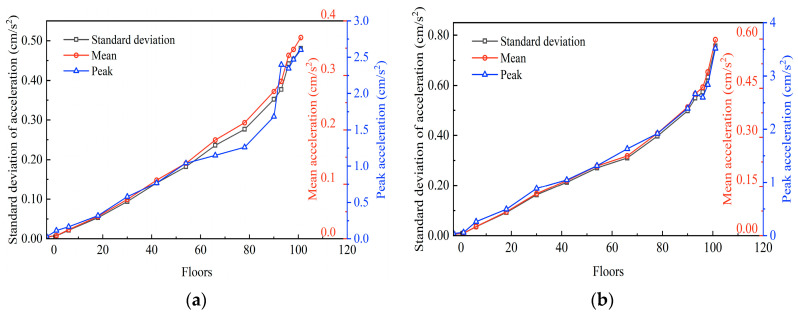
Mean accelerations, peak accelerations, and standard deviations of acceleration responses: (**a**) in the X−direction, and (**b**) in the Y−direction.

To further investigate the relationship between the acceleration amplitude and floor height, based on the measured results, the standard deviation and peak and mean values of the acceleration responses as the functions of the floor height are given in Figure 6. As shown, they follow similar trends with the increasing floor height in both X and Y directions. The maximum acceleration appeared on the 101st floor, demonstrating that the vibration increases with the structure height, and the dispersion of the acceleration signals becomes progressively greater.

### 4.3. Probability Density Function of Acceleration

**Figure 7:**
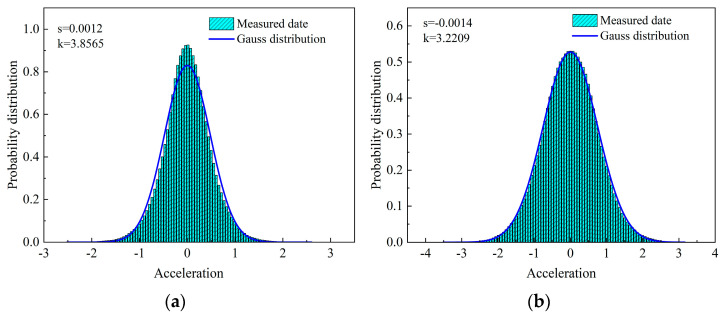
Acceleration probability density distributions: (**a**) in the X−direction, and (**b**) in the Y−direction.

The probability density functions (PDFs) of the measured vibration signals are calculated and shown in Figure 7. The graphs show that the acceleration signals have kurtosis coefficients of K = 3.8565 in the X direction and K = 3.2209 in the Y direction. They are compared with the Gaussian distribution [37]: when the skewness coefficient S > 0, the PDF is right-biased; while the skewness coefficient S < 0, the PDF is left-biased; when the kurtosis coefficient K > 3, the PDF is highly peaked; while the kurtosis coefficient K < 3, the PDF is lowly peaked. As seen, the present PDFs are highly peaked [38,39,40], which means the probability of strong vibration occurring on high floors is large, and thus, more attention should be paid to structural design and health monitoring.

### 4.4. Power Spectrum of Accelerations

The power spectral density of the time-domain acceleration signals is obtained using the mean periodogram method. This method involves segmenting the response signal data without overlapping and then averaging the power spectrum of individual segments [24].

**Figure 8:**
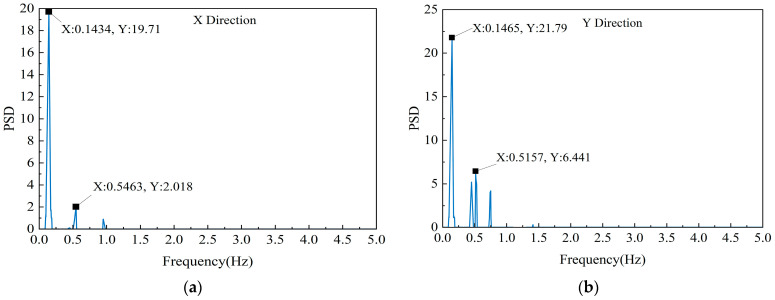
Power spectra of accelerations: (**a**) in the X−direction, and (**b**) in the Y−direction.

As shown in Figure 8, the spectra are clear and distinct, with the peaks neatly arranged in a regular pattern. The building’s wind-induced vibrations are highly consistent in both directions, and the first-order modes play a significant role. In the X direction, the first-order natural frequency is 0.143 Hz and the second-order natural frequency is 0.546 Hz; in the Y direction, the first- and second-order natural frequencies are 0.146 Hz and 0.516 Hz, respectively. The difference in the first-order natural frequency between the two directions is 0.003 Hz, and the difference in the second-order natural frequency is 0.03 Hz.

## 5. Parameter Identification Results

The natural frequencies, damping ratios, and mode shapes are the most basic parameters of structural dynamic properties, so accurate estimation of these three types of properties in structural design is of great importance [41]. In this section, the curve method and the standard deviation method are used to analyze the time history of accelerations of the 101st floor at the top of the SWFC in the X and Y directions.

### 5.1. Variation of Natural Frequencies and Damping Ratios with Amplitudes

**Figure 9:**
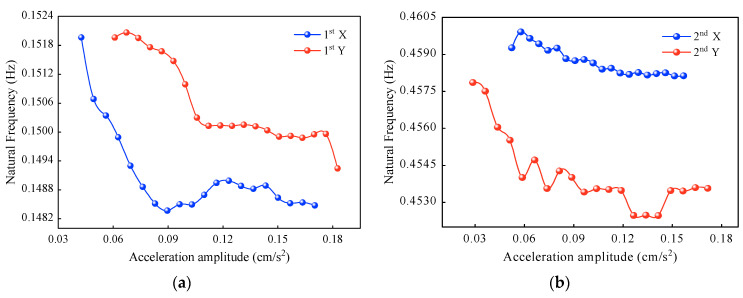
Natural frequencies determined by the curve method: (**a**) first-order natural frequencies, and (**b**) second-order natural frequencies.

**Figure 10:**
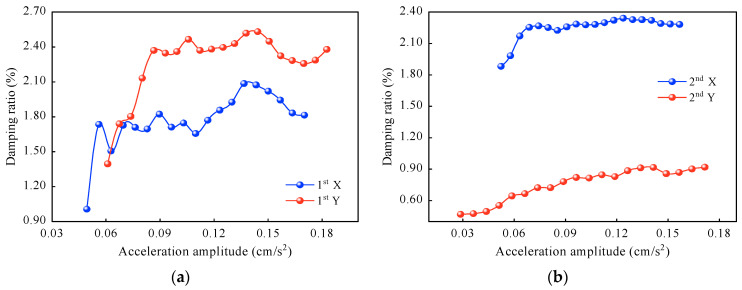
Damping ratios determined by the curve method: (**a**) first-order damping ratios, and (**b**) second-order damping ratios.

The first method is the curve method. Using the same acceleration time history, set 20 intercepted amplitudes in the range of 0.5~1.5 times the standard deviation to obtain the natural frequencies and damping ratios of the SWFC. Figure 9 and Figure 10 show the correlation curves of the first two natural frequencies and damping ratios with amplitudes, respectively.

As shown in Figure 9, the first- and second-order natural frequencies in both X and Y directions decrease as the amplitudes increase. The main reason is that nonlinear responses occur when structural amplitudes increase, and the steel joints slip, which makes the interactions between the structural and nonstructural elements greater. The natural frequencies identified by the curve method fluctuate considerably in the low-amplitude region, and the dependence becomes progressively more apparent as the amplitudes increase. The general trends are as follows: when the X-direction acceleration amplitudes increase from 0.04 cm/s^2^ to 0.17 cm/s^2^, the first-order natural frequency decreases by 0.0035 Hz, and the second-order natural frequency decreases by 0.0012 Hz; when the Y-direction acceleration amplitudes increase from 0.03 cm/s^2^ to 0.18 cm/s^2^, the first-order and second-order natural frequencies decrease by 0.0027 Hz and 0.0043 Hz, respectively. The maximum drop is 2.303% and the minimum drop is 0.261%, which are the first- and second-order natural frequencies in the X direction, respectively.

As shown in Figure 10, the first- and second-order damping ratios in the X and Y directions increase with the increasing amplitudes. The damping ratios identified by the curve method fluctuate considerably in the low-amplitude region, and their dependence increases with the increasing amplitudes. At the same time, in the high-amplitude region, the damping ratios tend to stabilize in different directions and even tend to increase in one direction. When the X-direction acceleration amplitudes increase from 0.04 cm/s^2^ to 0.17 cm/s^2^, the first-order damping ratio increases by 0.82%, and the second-order damping ratio increases by 0.40%. When the Y-direction acceleration amplitudes increase from 0.03 cm/s^2^ to 0.18 cm/s^2^, the first-order damping ratio increases by 0.98%, and the second-order damping ratio increases by 0.45%. The second-order damping ratio in the Y direction increases at most by 95.7%.

**Figure 11:**
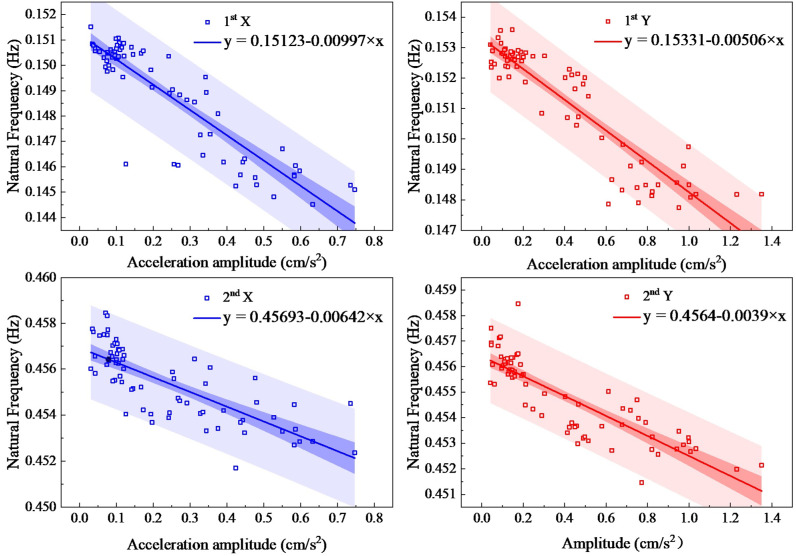
Natural frequencies determined by the standard deviation method.

**Figure 12:**
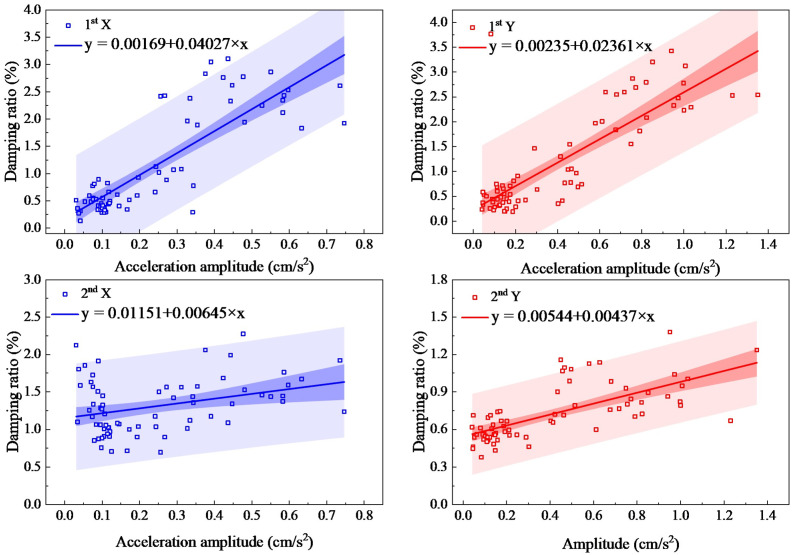
Damping ratios determined by the standard deviation method.

The second method is the standard deviation method. On the other hand, by setting the time interval as one hour and the standard deviation of the accelerations as amplitudes, structural frequencies and damping ratios are obtained. Figure 11 and Figure 12 show the trends of the first- and second-order natural frequencies and damping ratios with amplitudes for the standard deviation method, respectively. Equations (1) and (2) are used to make a linear fitting, and the fitting parameters are shown in Table 1.

(2) $$f=\alpha_{0}+\beta_{0}x$$

(3) $$\xi =\alpha_{1}+\beta_{1}x$$

where f is the natural frequency, ξ is the damping ratio, $\alpha_{0}$ and $\alpha_{1}$ are the natural frequency and damping ratio when the amplitude *x* is 0, respectively, and $\beta_{0}$ and $\beta_{1}$ are the change rates of the natural frequency and damping ratio with amplitude *x*, respectively.

As shown in Figure 11, the first- and second-order natural frequencies in both directions decrease as the amplitudes increase. From the linear fitting, it can be seen that the first-order natural frequency is well fitted and the scatter points lie within the 95% prediction band. The natural frequencies identified by the standard deviation method are concentrated in the low-amplitude region, and the amplitude dependence diminishes as the amplitudes increase. The general trends are as follows: when the X-direction acceleration amplitudes increase from 0.03 cm/s^2^ to 0.75 cm/s^2^, the first-order natural frequency decreases by 0.007 Hz, and the second-order natural frequency decreases by 0.0212 Hz; when the Y-direction acceleration amplitudes increase from 0.04 cm/s^2^ to 1.35 cm/s^2^, the first-order and second-order natural frequencies decrease by 0.0089 Hz and 0.007 Hz, respectively. The maximum drop is 5.794% and the minimum drop is 1.527%, which are the first- and second-order natural frequencies in the Y direction, respectively.

As shown in Figure 12, the first- and second-order damping ratios in both directions increase with increasing amplitude. It can be seen that the identified results of second-order damping ratios have a large discretization and a wide range of prediction bands. The first-order damping ratio is less scattered, which is identical with the measurements of the Guangzhou Tower by the Hong Kong Polytechnic University [42]. The general trends are as follows: when the X-direction amplitudes increase from 0.03 cm/s^2^ to 0.75 cm/s^2^, the first-order damping ratio increases by 2.86%, and the second-order damping ratio increases by 0.46%; when the Y-direction amplitudes increase from 0.04 cm/s^2^ to 1.35 cm/s^2^, the first-order damping ratio increases by 3.25%, and the second-order damping ratio increases by 0.62%.

The natural frequencies and damping ratios identified by the curve method and the standard deviation method are compared with other results in the literature. The comparisons are shown in Table 2 and Table 3. It was found that the two methods produced comparable results. Nonetheless, the dispersion of natural frequencies derived from the standard deviation method is large, the regularity is obscure, and the variation of the acceleration amplitudes is small. In contrast, the dispersion of the natural frequencies derived via the curve method is small, but the acceleration amplitude varies greatly. In addition, the first two orders of natural frequencies in the X and Y directions derived by the two methods are highly consistent with the calculations by other researchers. However, the first two orders of damping ratios are different. The first two orders of damping ratios derived from the curve method differ from the results of the three scholars by a maximum of 2.178 Hz, while the results derived from the standard deviation method fluctuate more.

### 5.2. Variation Patterns of the Natural Frequencies and Damping Ratios with the Mean Wind Speed

**Figure 13:**
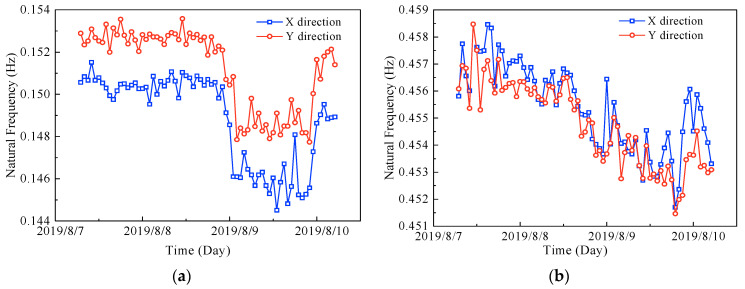
Variations of the natural frequencies with the mean wind speed: (**a**) first-order natural frequencies, and (**b**) second-order natural frequencies.

**Figure 14:**
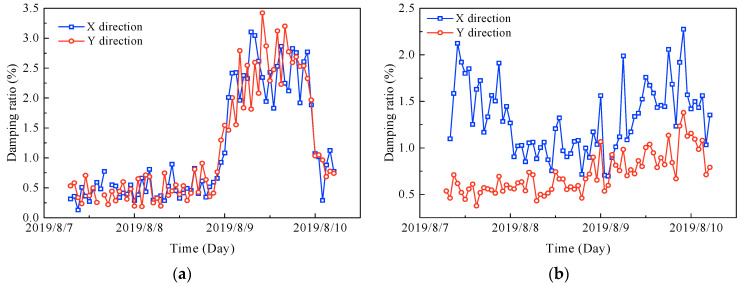
Variations of the damping ratios with the mean wind speed: (**a**) first-order damping ratios, and (**b**) second-order damping ratios.

On the basis of the measured results, the relationships of the natural frequencies and damping ratios with the mean wind speed are also found, as shown in Figure 13 and Figure 14. Comparing the mean wind speed (Figure 4a) with Figure 13 and Figure 14, the following summary can be obtained.

(1) The trends of the natural frequencies in the X and Y directions are basically the same, and both show negative correlations with the mean wind speed, i.e., the natural frequencies decrease as the mean wind speed increases, and the trend of the first-order natural frequencies is more pronounced.

(2) The damping ratios show a positive correlation with the mean wind speed, i.e., the damping ratios increase as the mean wind speed increases, except for the second-order damping ratio in the X direction, which may be due to the non-Gaussian nature of the original acceleration signals.

### 5.3. Integral Mode Shapes of the Structure

**Figure 15:**
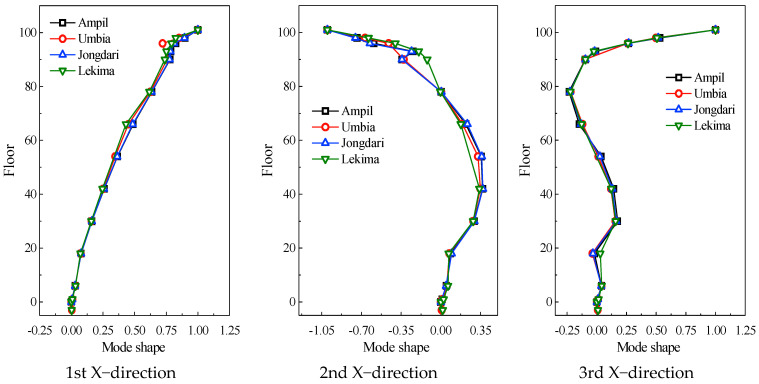

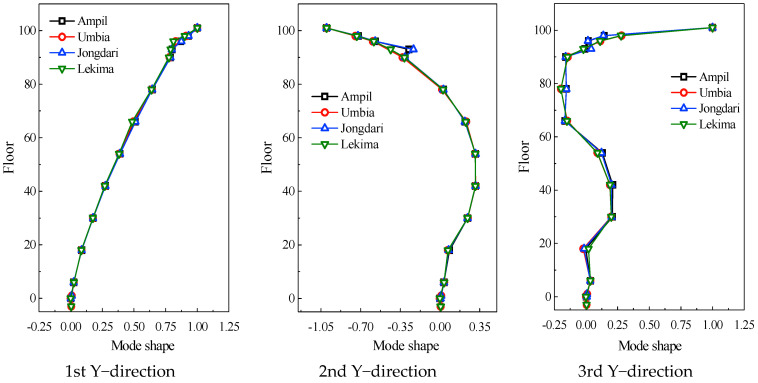
Comparisons of the mode shapes of the SWFC.

For integral mode shape identification, the frequency domain approach (FDA) is utilized, i.e., the ratio of the mutual spectrum to the self-spectrum of the acceleration signal is used as an approximation to determine the ratio of mode shapes [45]. The mode shapes on the 101st floor are used for normalization to obtain the first three orders of mode shapes. The results are also compared with those excited by the other three different typhoons, i.e., Ampil [30], Rumbia [30], and Jongdari [46], as shown in Figure 15.

As seen, the mode shapes of the SWFC were essentially the same under Typhoons Lekima, Ampil, Rumbia, and Jongdari. The first-order mode shape has one zero point, the second-order has two, and the third-order has three. Both the X- and Y-directional mode shapes present an increasing nonlinear trend with the increasing floor level, especially the third-order mode shapes. The X-direction and Y-direction trends are virtually the same, indicating that the difference between the stiffness of the SWFC in the X direction and Y direction is not substantial, which complies with the findings of the natural frequency.

## 6. Conclusions

Based on the measured wind-induced vibration responses of the SWFC under Typhoon Lekima, this paper studies the amplitude correlations and variation patterns of the dynamic parameters, and the following main conclusions are obtained:

(1) The wind-induced vibration responses in the X and Y directions are highly consistent. The maximum acceleration response occurs in the Y direction, which is about 3.518 cm/s^2^, and the maximum instantaneous acceleration in the X direction is 2.602 cm/s^2^, both of which meet the design requirements.

(2) The curve method and the standard deviation method are effective and accurate in identifying dynamic characteristic parameters. The first-order natural frequency in the X direction is about 0.151 Hz, and the one in the Y direction is about 0.153 Hz; the second-order natural frequency in both X and Y directions is about 0.46 Hz. The first- and second-order damping ratios in the X and Y directions are less than 1%, which may be related to the dampers installed in the SWFC.

(3) The first- and second-order natural frequencies in both the X and Y directions decrease with the increasing amplitudes; the first- and second-order damping ratios in both the X and Y directions increase with the increasing amplitudes. In addition, it is found that the natural frequencies show negative correlations with the mean wind speed, while the damping ratios show positive correlations.

(4) The mode shapes of the SWFC are essentially the same under different typhoon excitations. The first- and second-order mode shapes in the X and Y directions grow with the increase in floor levels, displaying a nonlinear trend, and the third-order mode shapes have more obvious nonlinear trends.

## Figures and Tables

**Table 1:** Fitting parameters of the curve method.

Fitting Parameters	First Order		Second Order	
	X Direction	Y Direction	X Direction	Y Direction
Natural frequencies	*α*_0_ = 0.15123, *β*_0_ = −0.00997, R^2^ = 0.796	*α*_0_ = 0.15331, *β*_0_ = −0.00506, R^2^ = 0.836	*α*_0_ = 0.45693, *β*_0_ = −0.00642, R^2^ = 0.6	*α*_0_ = 0.4564, *β*_0_ = −0.0039, R^2^ = 0.724
Damping ratios	*α*_1_ = 0.00169, *β*_1_ = 0.04027, R^2^ = 0.69	*α*_1_ = 0.00235, *β*_1_ = 0.02361, R^2^ = 0.66	*α*_1_ = 0.01151, *β*_1_ = 0.00645, R^2^ = 0.33	*α*_1_ = 0.00544, *β*_1_ = 0.00437, R^2^ = 0.47

(R^2^, also known as the coefficient of determination of the equation, indicates the degree to which the variable X in the equation explains Y. R^2^ values are between 0 and 1, and the closer to 1, the better X explains Y in the equation.)

**Table 2:** Comparison of the natural frequencies under the excitation of Typhoon Lekima.

Method or Study	First-Order Natural Frequencies (Hz)		Second-Order Natural Frequencies (Hz)	
	X Direction	Y Direction	X Direction	Y Direction
Standard deviation method	0.1512	0.1533	0.4569	0.4564
Curve method	0.1519	0.1521	0.4600	0.4579
Ya Jun Huang [43]	0.1513	0.1535	/	/
Yun Cheng He [44]	0.1510	0.1530	0.4660	0.4660
Zeng shun Chen [45]	0.1511	0.1526	/	/

**Table 3:** Comparison of the damping ratios under the excitation of Typhoon Lekima.

Method or Study	First-Order Damping Ratios (%)		Second-Order Damping Ratios (%)	
	X Direction	Y Direction	X Direction	Y Direction
Standard deviation method	0.13~3.104	0.19~3.77	0.70~2.28	0.38~1.38
Curve method	2.087	2.532	2.341	0.917
Ya Jun Huang [43]	3.349	3.778	/	/
Yun Cheng He [44]	2.310	0.354	2.090	0.753
Zeng shun Chen [45]	0.651	0.688	/	/

## Data Availability

All data generated or analyzed during this study are included in this article. All data included in this study are available upon request by contact with the corresponding author.
